# Global status, recent trends, and knowledge mapping of olive oil research and cardiovascular disease: 50 years of investigations through bibliometric analysis

**DOI:** 10.1002/fsn3.3885

**Published:** 2023-11-29

**Authors:** Baby Gargi, Sakshi Painuli, Prabhakar Semwal, Deependra Pratap Singh, Rohit Sharma, Abdur Rauf, Anees Ahmed Khalil, Ahood Khalid, Hassan A. Hemeg, Polrat Wilairatana

**Affiliations:** ^1^ Department of Biotechnology Graphic Era (Deemed to be University) Dehradun India; ^2^ Natural Products Research Laboratory Uttarakhand Council for Biotechnology (UCB) Dehradun India; ^3^ Research and Development Cell Graphic Era Hill University Dehradun India; ^4^ Department of Biotechnology Engineering, University Institute of Engineering Chandigarh University Chandigarh India; ^5^ Department of Chemistry University of Swabi Swabi Pakistan; ^6^ Faculty of Allied Health Sciences, University Institute of Diet and Nutritional Sciences The University of Lahore Lahore Pakistan; ^7^ Department of Medical Laboratory Technology, College of Applied Medical Sciences Taibah University Medina Saudi Arabia; ^8^ Department of Clinical Tropical Medicine, Faculty of Tropical Medicine Mahidol University Bangkok Thailand

**Keywords:** bibliometric analysis, cardiovascular diseases, olive oil, research trends, Scopus

## Abstract

In the Mediterranean diet, olive oil serves as the predominant fat source and has been linked to a decreased risk of mortality related to cardiovascular diseases (CVD). Still, there is no conclusive evidence correlating olive oil consumption to CVD. The aim of this study is to assess the global research, current research trends, and knowledge mapping related to the correlation between the consumption of olive oil and CVD using bibliometric analysis. On August 19, 2023, a title‐specific literature search was conducted on the Scopus database using the search terms “olive oil” and “cardiovascular disease” with a date range of the past 50 years. Subsequently, bibliometric tools such as VOSviewer and Bibliometrix were employed to analyze and evaluate the obtained documents. The search yielded (*n* = 429) publications and showed an upward trend in the annual publication count over the last five decades. The publication number exhibited a gradual increase with a rate of 5.55%. The results also indicated that 2530 authors, 759 institutions, 47 countries, and 223 journals have publications in this research domain. The present bibliometric study will be a valuable research reference for describing the worldwide research patterns concerning the relationship between olive oil and CVD during the past 50 years. In the future, the application of olive oil for the treatment of CVDs may be an emerging research trend. Apart from this, collaborations among authors, countries, and organizations are expected.

## Graphical abstract

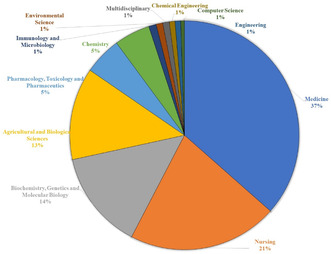

## 1 INTRODUCTION

Cardiovascular diseases encompass a range of heart and blood vessel disorders, such as coronary artery disease, cerebrovascular, peripheral artery, and rheumatic heart diseases (Olvera Lopez et al., 2021). In recent years, CVDs have been listed as the primary cause of global mortality and account for 17.9 million deaths annually. Over 80% of CVD‐related deaths result from heart attacks and strokes, with one‐third of these deaths occurring prematurely in individuals under 70 years of age (Cardiovascular diseases [https://www.who.int/health‐topics/cardiovascular‐diseases#tab=tab_1], accessed on August 20, 2023). Numerous dietary approaches have been proposed to address these concerns, including the Mediterranean diet, the stop‐hypertension diet, the vegetarian diet, and the ketogenic diet (Borges et al., 2017). The Mediterranean diet was recommended as a crucial intervention against CVDs in the 2015–2020 Dietary Guidelines for Americans (Lu et al., 2023). Olive oil (*Olea europaea*) serves as the root source of fat in the Mediterranean diet (Döding et al., 2023; Mazzocchi et al., 2019). Various processing techniques can produce different varieties of olive oil, including virgin, refined, and standard olive oil (Ruiz‐Canela & Martínez‐González, 2011). The categorization of olive oil relies on sensory attributes such as aroma and flavor, as well as physiochemical traits, including peroxide value and free acidity (Mehmood et al., 2020). The consumption of olive oil has been observed to exhibit an inverse correlation with the overall mortality rate due to cardiovascular events (Covas, 2007; Xia et al., 2022). The connection might be more robust for virgin olive oil and could potentially have an impact from on the initial stages of the CVDs (Donat‐Vargas et al., 2022). In terms of its nutritional composition, triglycerides make up about 98% of the neutral lipids in olive oil, while 1%–2% are polar lipids, which are made up of a broad range of complex chemical constituents (Antonopoulou & Demopoulos, 2023). Olive oil also comprises a saponifiable and unsaponifiable fraction, which corresponds to its fatty acid composition, primarily consisting of monounsaturated fatty acids (Marcelino et al., 2019; Schwingshackl & Hoffmann, 2014). Although there are some comprehensive articles related to the impact of olive oil consumption and prevention of CVDs, their studies involved only specific aspects (Nocella et al., 2018; Visioli et al., 2018; Zampelas & Kafatos, 2004). However, no definitive link has been established between olive oil consumption and CVDs (Donat‐Vargas et al., 2022; Guasch‐Ferré et al., 2014). Hence, through the utilization of bibliometric analysis, our aim was to understand the current research trends, influential sources, articles, authors, countries, and hotspots in this domain.

Bibliometric analysis is a widely accepted and precise approach to investigating and scrutinizing vast amounts of scientific literature. Through the process, we have the ability to explore the detailed facts of a particular discipline and gain insight into the developing aspects within that field (Donthu et al., 2021). Therefore, in the present study, we outline the primary contributions to the recent scientific literature on the recent trends of olive oil research and CVDs by performing a bibliometric analysis.

### 1.1 Research review approach

By employing a question‐based approach, we aim to enhance understanding and further exploration in this field. These contributions are framed as a series of questions, as follows:

**Question 1:** What were the dynamic changes in research trends of olive oil and CVD, based on publication distribution?

The exploration into the dynamic evolution of olive oil research and its correlation with CVD based on publication distribution yields valuable insights into the overarching trajectory and advancements over time. These insights are of great utility to researchers seeking to deepen their understanding of this field.

**Question 2:** Which countries, institutions, and authors exerted the greatest influence and demonstrated the highest level of productivity in the advancement of olive oil research and CVD?

The examination of this matter aims to identify the countries, institutions, and authors that exerted the greatest influence and demonstrated the highest level of productivity in the advancement of olive oil research and CVD. This analysis is also expediting the search for potential collaboration opportunities.

**Question 3:** Which journals have made the greatest contributions to research based on olive oil and CVD?

Examining the most productive journals for publishing relevant research work is a crucial consideration for researchers. This paper presents a systematic analysis that synthesizes the theoretical and empirical findings to highlight the development of research trends in olive oil and CVD, along with the journals that have demonstrated the highest productivity in this field.

**Question 4:** What is the trajectory of development and the current emerging trends in olive oil research and CVD?

This response provides substantial assistance to all communities engaged in the study of this field by facilitating their access to the latest developments and emerging trends within this domain. Additionally, it also highlights the potential approaches for future research. To achieve this, various analytical techniques, such as keyword co‐occurrence, clustering, and timeline analysis, are employed to offer valuable insights into the current state of research and exploration within this domain.

The present paper is organized in a systematic manner, commencing with Section 2, which outlines the methodology employed to conduct this study. Following that, Section 3 presents the results and discussion of the bibliometric analysis conducted on the collected dataset. The limitation of this study is discussed in Section 4. In Section 5, the existing gaps in the literature are identified, along with potential opportunities for further research on the topic. Finally, Section 6 provides the complete conclusions drawn from the study.

## 2 METHODOLOGY

The aim of this review is to establish an extensive foundation comprising distinct stages for systematically reviewing prior research on the correlation between olive oil and CVD and then performing a bibliometric analysis. The fundamental sections of the designed framework comprise (i) identification and collection of relevant data and (ii) bibliometric analysis of the data. A detailed explanation of each stage is provided in the subsequent sub‐sections.

- Identifying and collecting the data

**FIGURE 1:**
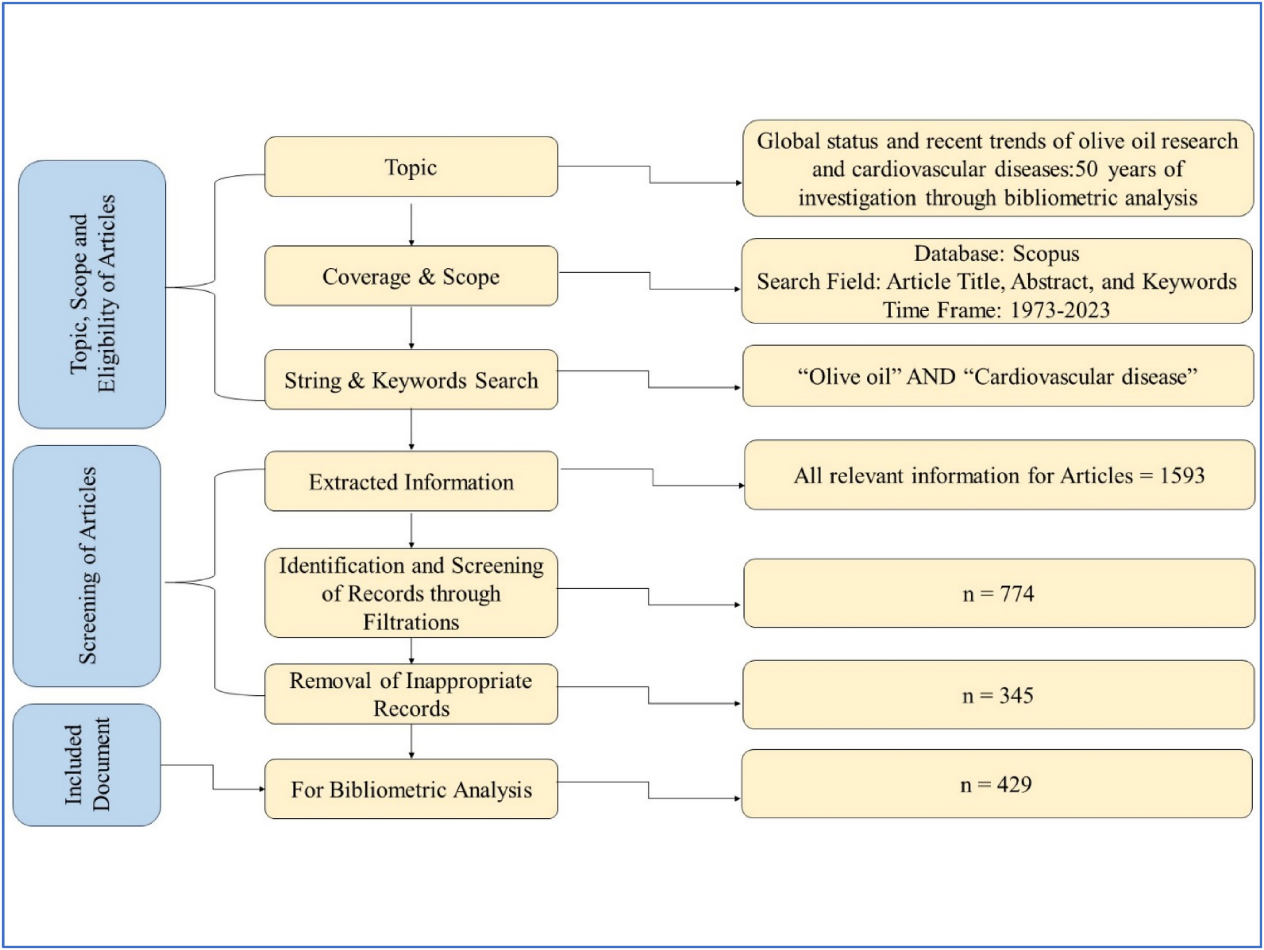
Visualization of the search methodology as a flowchart.

In order to search relevant literature pertaining to olive oil research and CVDs within a comprehensive framework, the following criteria were employed: “*TITLE‐ABS‐KEY” = “Olive oil” AND “Cardiovascular diseases”; “PUBYEAR” = “1973–2023”; “LIMIT‐TO” = PUBSTAGE, “final”; DOCTYPE, “ar”; LANGUAGE, “English”*. The Scopus database is utilized for the purpose of conducting searches while considering the following limitations: (a) the search is conducted within the fields of “Title, Keywords, or Abstract”; (b) the time frame is restricted to the years between 1973 and 2023; (c) only publications in the English language are considered; (d) the document type is selected as “Articles” only; and (e) studies in the “Final” stage of publication were considered. The preliminary search yielded a total of 1593 documents. Subsequently, after applying the above‐mentioned limitations, only 774 documents were retrieved. Then, the abstracts, keywords, and titles of the articles were scrutinized, and the entire text was thoroughly screened to eliminate any instances of duplication or irrelevance. Therefore, after the second round, a total of 429 articles remained and were selected for further analysis. Figure 1 illustrates the steps followed in screening, selection, and analysis of data.

ii Analysis and visualization of data

In the current analysis, data extracted from the available literature were analyzed by employing Bibliometrix (version R.4.3.0) and VOSviewer (version 1.6.19). Bibliometrix is a software tool designed specifically for network analysis and visualization (Chen & Gong, 2022). Initially, bibliographic data sourced from the Scopus database was formatted in a [.csv] file. The Bibliometrix R package was subsequently installed and initialized in R Studio, and the Biblioshiny web application, which is designed for non‐programmers, was launched (Uyanga et al., 2023). The Biblioshiny application was utilized to generate visual representations of the density distribution of countries, organizations, and author collaboration, keywords, and trending topics (Mohammed & Li, 2023; Pritchard, 1969; Semwal et al., 2023).

Furthermore, we employed VOSviewer as a tool to conduct an analysis of bibliographic coupling among documents, co‐authorship between authors and countries, co‐occurrence among author keywords, and co‐citation analysis of cited references. The VOSviewer is an open‐source software tool developed by the Leiden University Centre for Science and Technology Studies for the mapping and visualization of bibliometric networks (Van Eck & Waltman, 2011). This methodology enabled researchers to visually explore connections and trends within academic literature efficiently.

## 3 RESULTS AND DISCUSSION

Table 1 displays key bibliometric data concerning the research focusing on olive oil and CVDs, obtained by using the Biblioshiny tool. From this data, we found 429 original research articles from 223 distinct sources. On average, these articles exhibit an average citation rate of 72.11, showing the significant impact of the accumulated research in this domain.

**TABLE 1:** Summary of the key information.

Description	Results
Timespan	1987:2023
Sources (Journals, Books, etc.)	223
Documents	429
Annual Growth Rate %	5.55
Document Average Age	10.1
Average Citations Per Doc	72.11
References	20,369
Keywords Plus (ID)	3677
Author's Keywords (DE)	1015
Authors	2530
Authors of Single‐Authored Docs	17
Co‐Authors Per Doc	8.99
International Co‐Authorships %	21.91
Document Types (Article)	429

### 3.1 Annual publication growth and citation

**FIGURE 2:**
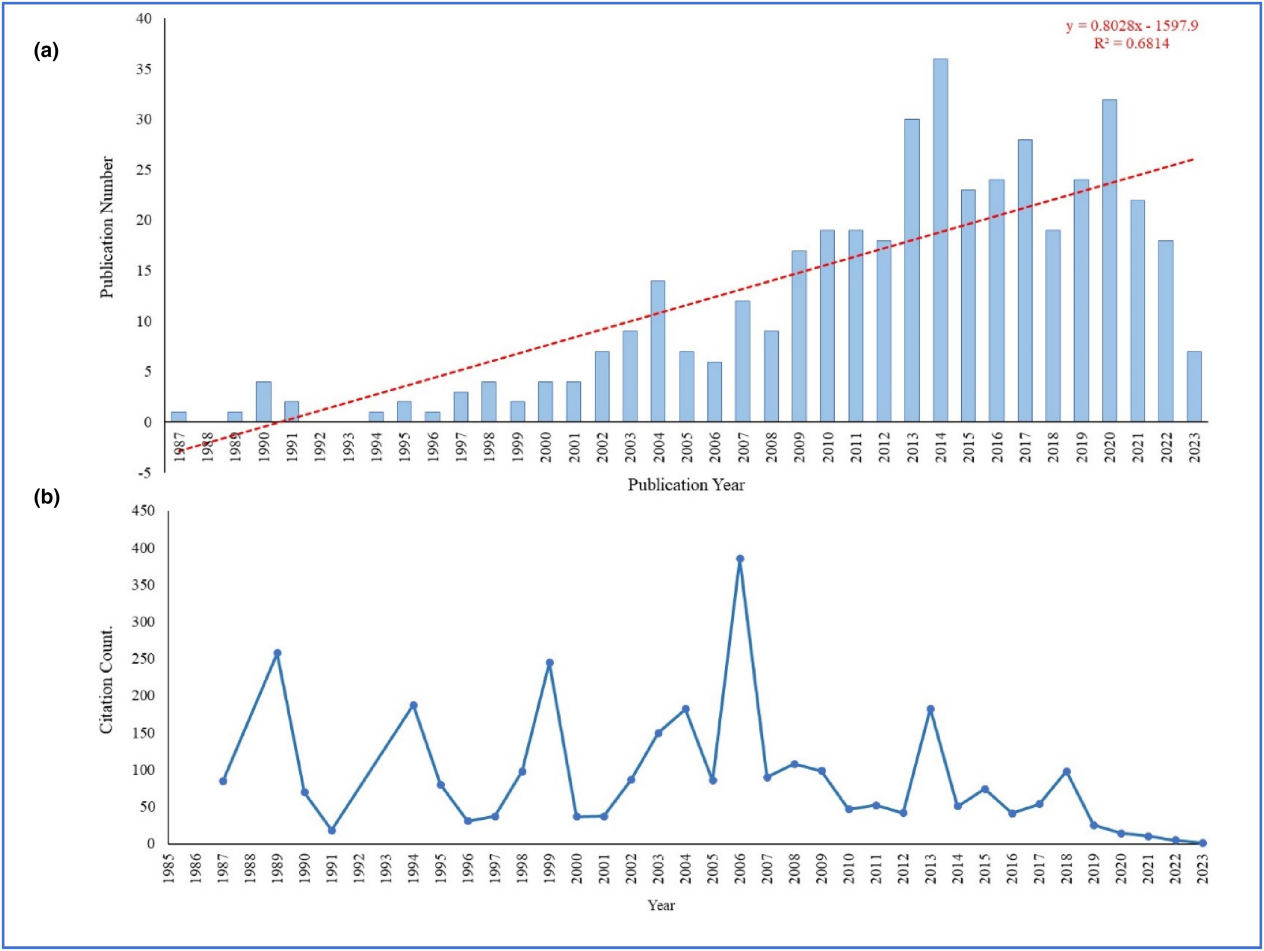
(a) Annual count of published articles; (b) Annual mean total citation count per document.

The annual publication count can indicate the progress and knowledge growth in the corresponding research field (Yang et al., 2023). Figure 2 depicts the annual publication number, trends, and average citations from 1987 to 2023 (August 19, 2023) within the field of olive oil research and CVDs. Over the past 50 years, the evolution of this field can be categorized into three distinct stages, i.e., the initial phase (1987–2021), the transitional phase (2002–2008), and the exponential phase (2009–2023). The annual number of publications grew steadily, with an annual growth rate of 5.55% and an average citation of 72.11 per article. The literature output exhibited a linear growth curve, as evidenced by the fitting curve function equation y = 0.8028x − 1597.9, with a *R* ^2^: .6814. Moreover, there is an upward trend in the present trajectory, indicating that research related to the impact of olive oil on CVDs is undergoing a phase of consistent advancement. It is probable that a considerable amount of research will emerge in the forthcoming period.

### 3.2 Distribution of output in subject categories

**FIGURE 3:**
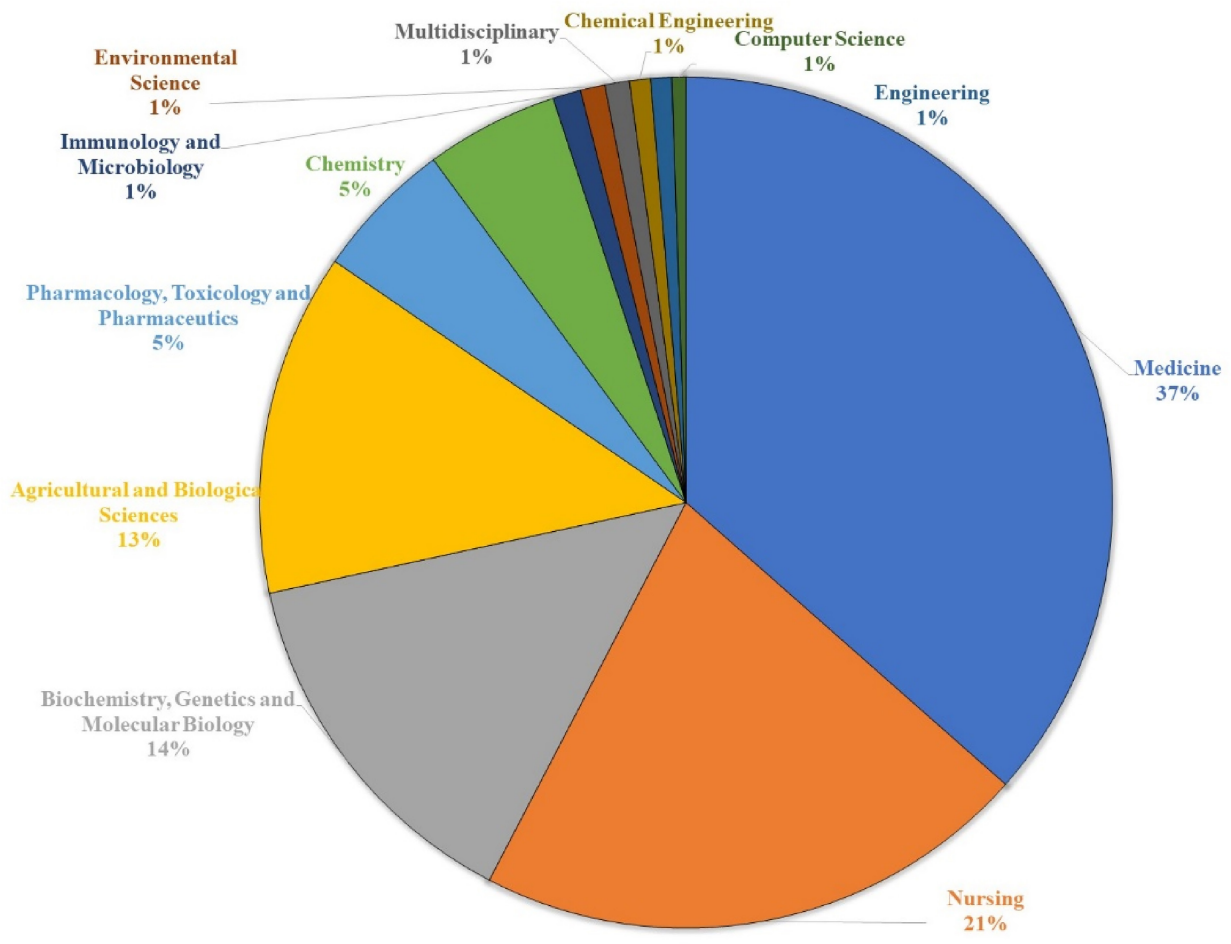
Map depicting the distribution of discipline categories.

The study of olive oil's impact on CVDs encompasses various fields, resulting in a vast distribution of research subjects. Figure 3 illustrates the distribution of disciplinary categories within the scope of this research area. The majority of the publications were related to the discipline of Medicine, followed by Nursing, Biochemistry, Genetics, and Molecular Biology.

### 3.3 Most productive countries/regions by corresponding authors

**FIGURE 4:**
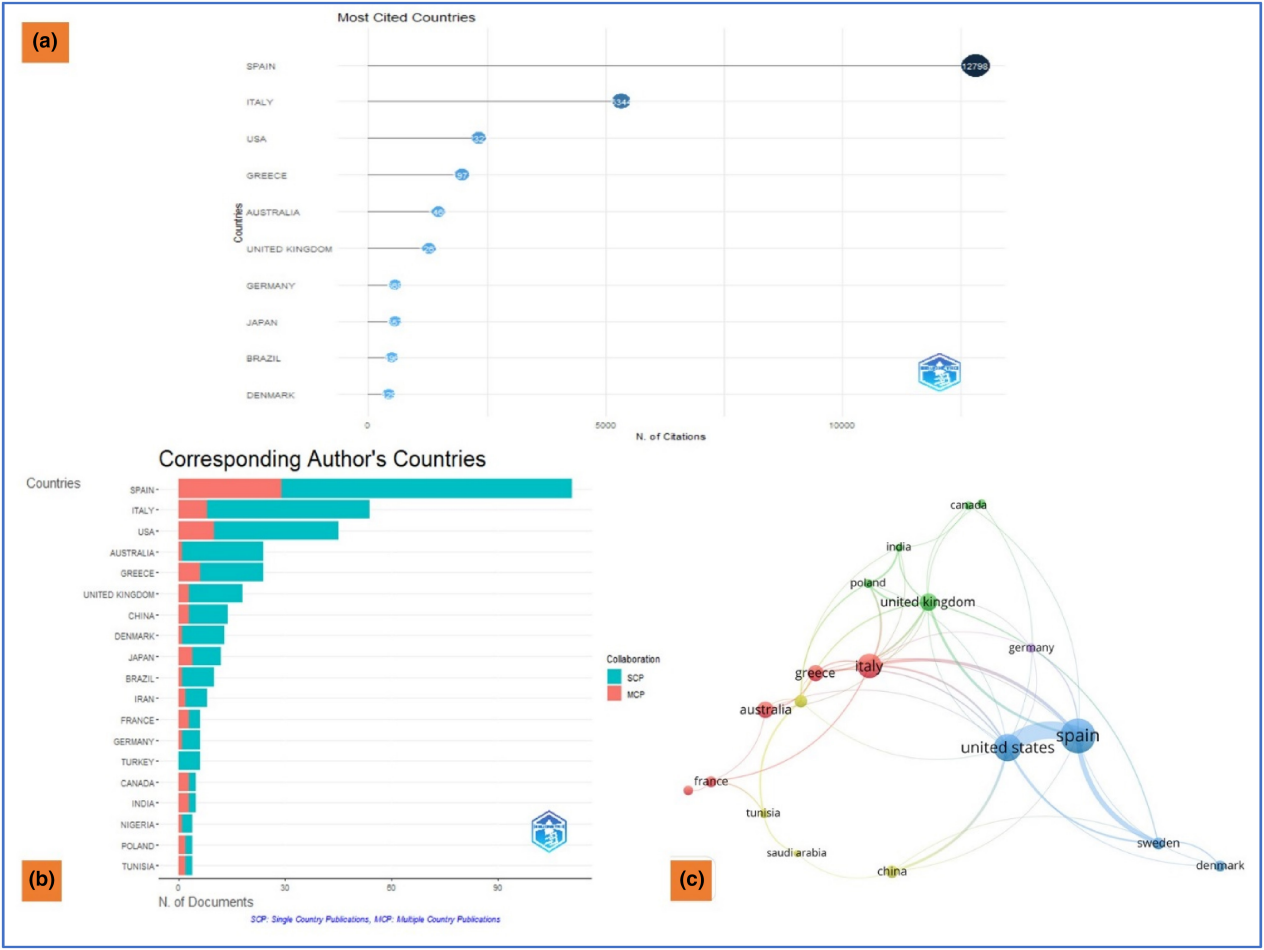
(a) Most cited countries; (b) Single and multiple country publications of the corresponding author's countries; (c) Network map visualization of the co‐authorship among countries among documents on olive oil research and cardiovascular diseases.

This section highlights the most influential and productive countries. Over the span of the last 50 years, a total of 40 countries have published articles in this discipline. In terms of research productivity, Spain is the leading country with a publication count of 111, followed by Italy and the USA. The top ten ranked countries in terms of citations are listed in Figure 4a. However, the collaboration among single (SCP) and multiple country participation (MCP) is presented in Figure 4b and Table 2. Figure 4c illustrates the network analysis map of co‐authorship among countries. VOSviewer analysis revealed that out of 40 countries, 21 meet the threshold value of containing a minimum of five documents. The result yielded five clusters of Red (5), Green (5), Blue (4), Yellow (4), and Violet (1) number, respectively.

**TABLE 2:** List of top 10 most productive countries by corresponding authors.

Rank	Country	Articles	SCP	MCP	Frequency	MCP_Ratio
1	Spain	111	82	29	0.25874126	0.261261261
2	Italy	54	46	8	0.12587413	0.148148148
3	USA	45	35	10	0.1048951	0.222222222
4	Australia	24	23	1	0.05594406	0.08
5	Greece	24	18	6	0.05594406	0.166666667
6	United Kingdom	18	15	3	0.04195804	0.214285714
7	China	14	11	3	0.03263403	0.214285714
8	Denmark	13	12	1	0.03030303	0.076923077
9	Japan	12	8	4	0.02797203	0.333333333
10	Brazil	10	9	1	0.02331002	0.1

### 3.4 Most relevant institutions/organizations

The present section explores the geographical distributions of the affiliations of authors. Through organization analysis, we can examine the most actively participating institutions in the field of olive oil and CVD research trends. Table 3 represents the top 10 research institutions based on their production. The “*University of Navarra*” occupies the first position with 107 published articles, followed by the “*University of Barcelona*”, “*Instituto de Salud Carlos*”, “*University of Valencia*”, and “*Reina Sofia University Hospital*”.

**TABLE 3:** List of the top 10 productive organizations.

Rank	Affiliation	Articles
1	University of Navarra	107
2	University of Barcelona	100
3	Instituto De Salud Carlos	87
4	University of Valencia	69
5	Reina Sofia University Hospital	51
6	Institut D'investigacions Biomèdiques August Pi I Sunyer	40
7	University of Las Palmas De Gran Canaria	39
8	Harokopio University	36
9	University Rovira I Virgili	36
10	Sapienza University of Rome	34

### 3.5 Authors’ contribution analysis

**FIGURE 5:**
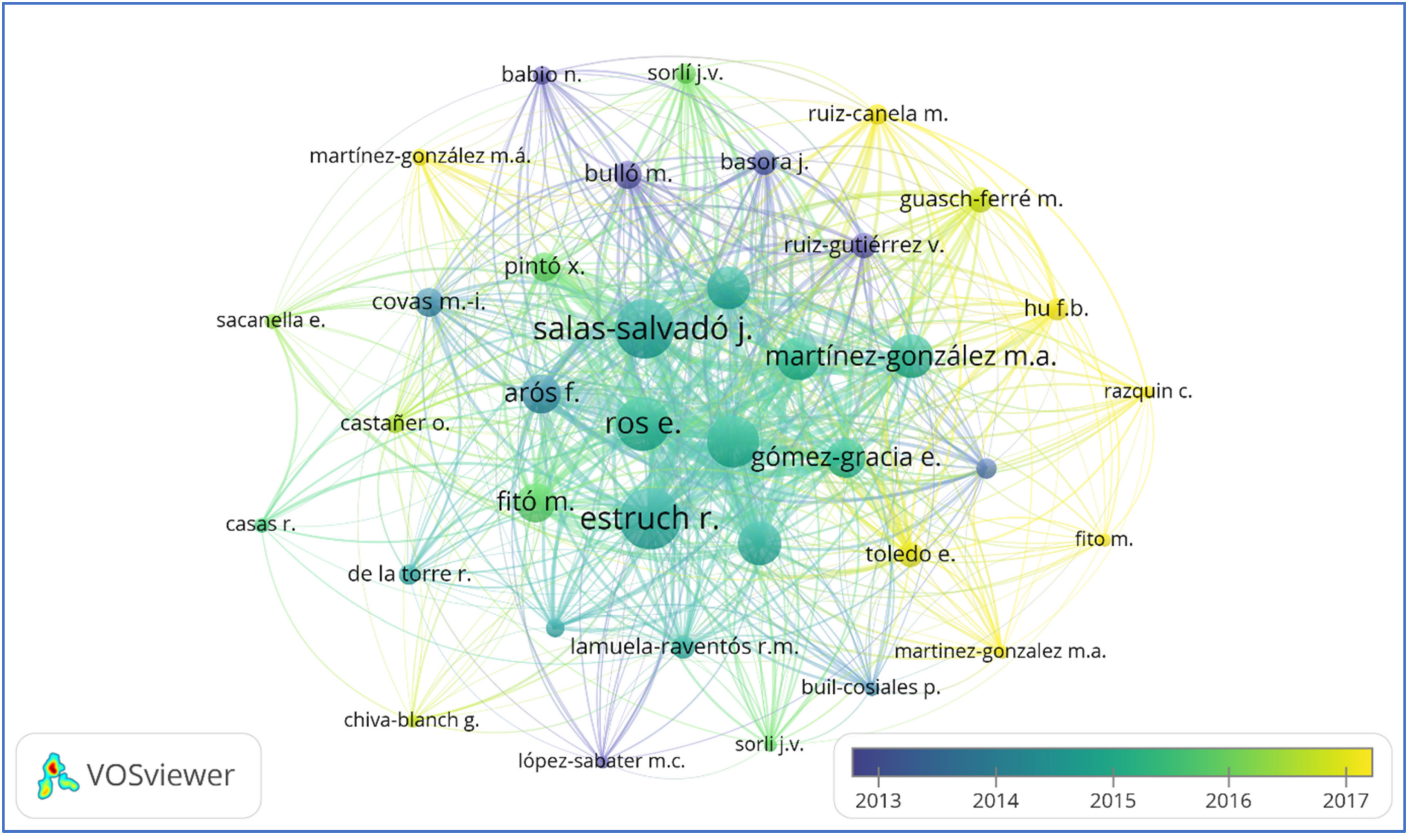
Co‐authorship among authors.

The number of scientific works published by an author can serve as a measure of their involvement and impact within their field of study (Zhou et al., 2022). The details of the most influential authors according to their productivity are listed in Table 4. A total of 2181 authors were included in 429 publications. As per the result obtained, Estruch R. was the most influential author with the maximum number of articles. A collaboration network analysis of authorship among authors was generated using VOSviewer. The overlay visualization map of co‐authorship is presented in Figure 5. The nodes represent the authors, and their size indicates the number of articles they published (Yang et al., 2023).

**TABLE 4:** List of the top 10 authors based on their publication quantity and contributions.

Rank	Authors	Publications	Articles fractionalized
1	Estruch R	49	3.68958
2	Salas‐Salvadó J	47	3.43698
3	Ros E	41	2.46331
4	Corella D	40	2.23744
5	Martínez‐González MA	31	1.65259
6	Serra‐Majem L	30	1.48698
7	Fiolm	29	1.49310
8	Fitó M	29	1.34690
9	Lapetra J	29	1.26739
10	Arós F	27	1.03086

### 3.6 Distribution of scientific productivity frequencies

**FIGURE 6:**
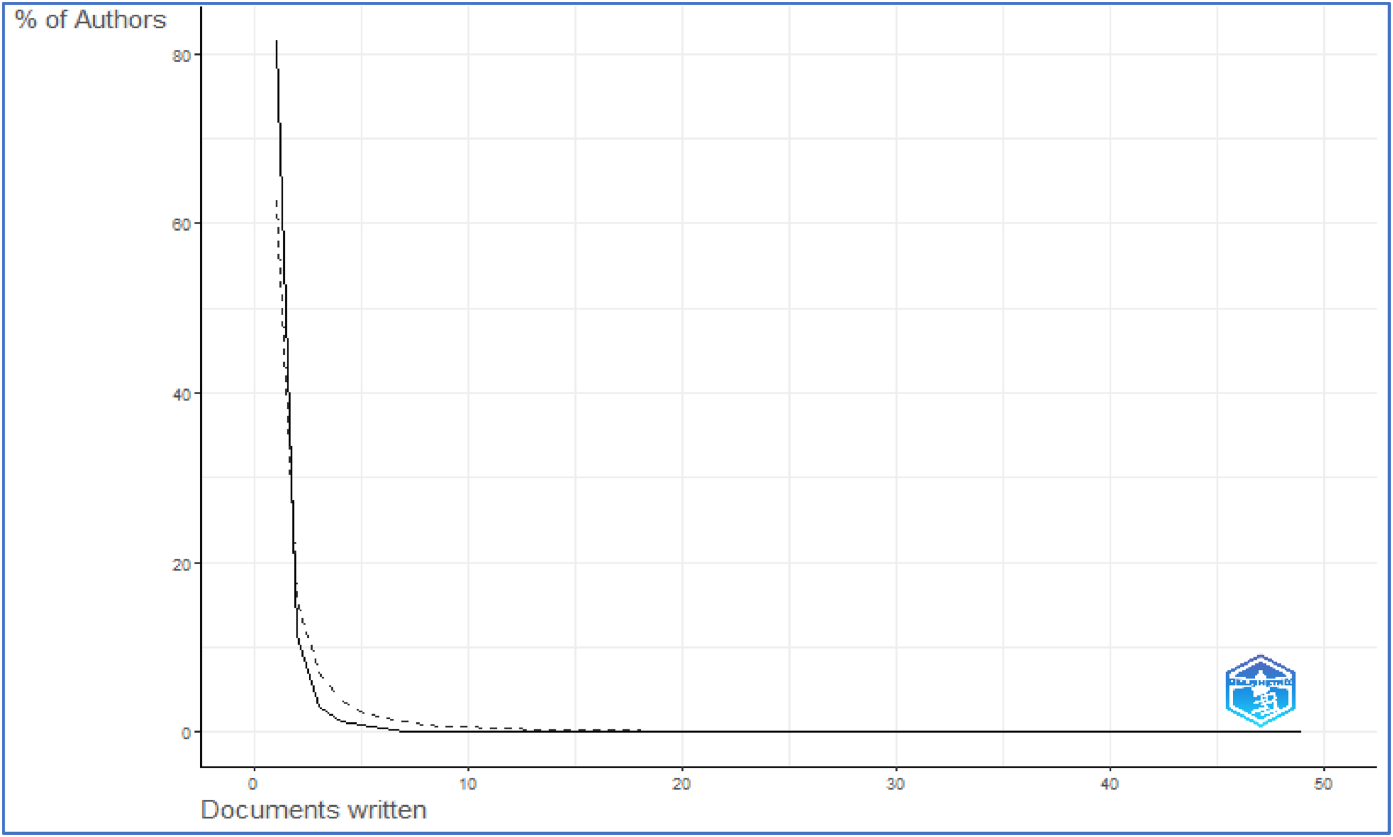
Distribution of scientific productivity based on Lotka's law.

Lotka's law is a theoretical construct that elucidates the correlation between authors and the number of published papers. This law is employed to demonstrate the manner in which authors are distributed over a time period or across specific disciplines (Franco et al., 2023). The present research field exhibits the concise implementation of Lotka's law, as shown by the distribution of author frequency and the publication count, as depicted in Figure 6. Table 5 illustrates the distribution of scientific productivity in accordance with Lotka's law.

**TABLE 5:** Analysis of scientific productivity distribution as per Lotka's law.

Documents written	Number of authors	Proportion of authors
1	2061	0.81462451
2	288	0.11383399
3	78	0.03083004
4	31	0.01225296
5	21	0.0083004
6	13	0.00513834
7	4	0.00158103
8	3	0.00118577
9	2	0.00079051
10	4	0.00158103

### 3.7 Most relevant sources

The efficacy of the most influential journal is determined by the number of its published articles and citations (Dzikowski, 2018). Consequently, we conducted an analysis of the number of articles associated with journals. According to the data extracted from the Scopus database, this study identified 429 articles published in 223 journals. The data revealed that the most productive journal with 21 documents is *Nutrients*, followed by the “*American Journal of Clinical Nutrition*” (*n* = 20) and “*Nutrition, Metabolism and Cardiovascular Diseases*” (*n* = 17). The list of the top 10 highly influential journals in the field of olive oil and CVD research is shown in Table 6.

**TABLE 6:** Top 10 most influential journals/sources.

Rank	Journal	Country	Impact factor	Number of articles
1	*Nutrients*	Switzerland	5.9	21
2	*American Journal of Clinical Nutrition*	United states	7.1	20
3	*Nutrition, Metabolism and Cardiovascular Diseases*	The Netherlands	3.9	17
4	*Journal of Nutrition*	United states	4.2	12
5	*Molecular Nutrition and Food Research*	Germany	5.2	11
6	*Atherosclerosis*	Ireland	5.3	9
7	*European Journal of Clinical Nutrition*	United kingdom	4.7	7
8	*European Journal of Nutrition*	Germany	5	7
9	*Clinical Nutrition*	United states	6.3	6
10	*PLoS One*	United states	3.7	6

**FIGURE 7:**
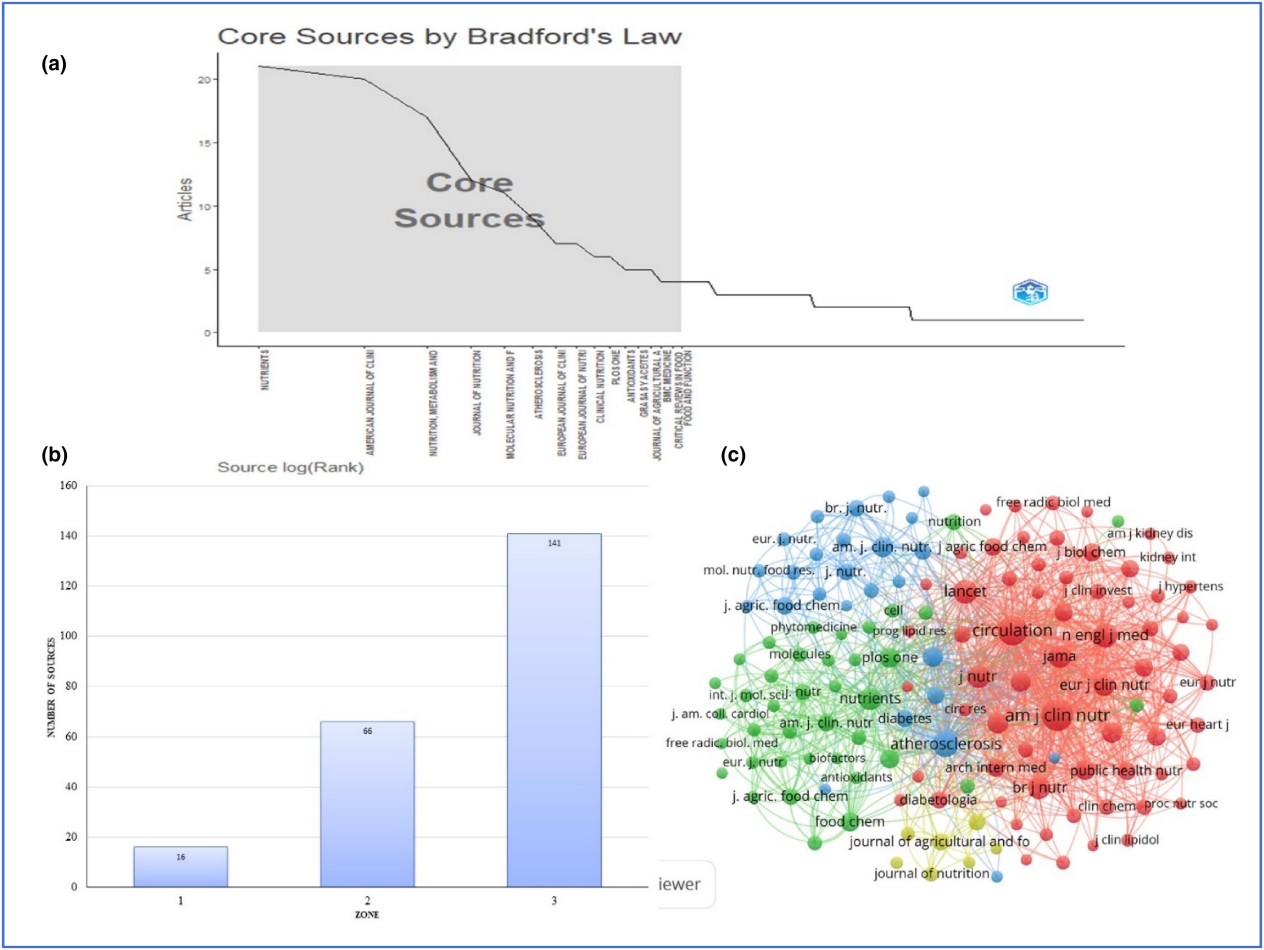
(a) Clustering of sources through Bradford's Law of Scattering; (b) Zone clustering of sources through Bradford's Law; (c) Visualization map of co‐citation among sources.

The clustering of these sources by Bradford Law is illustrated in Figure 7a. In 1934, S.C. Bradford proposed Bradford's law of scattering (Budd, 1988). This law has been used to determine the core sources in a specific domain; it describes the distribution pattern of journals in a given dataset (Patra et al., 2006). In accordance with this law, for this particular study, the publications in the field of olive oil and CVD research are almost equally distributed into three zones. Zone 1 consists of a small number of sources that account for one‐third of all publications. On the other hand, Zone 2 includes more journals, while Zone 3 has a larger number of journals. These zones consist of 16, 66, and 141 sources, respectively. As depicted in Figure 7b, it can be observed that out of the 429 publications in olive and CVD research, only 16 sources have contributed to one‐third of these publications. In terms of ranking within Zone 1, the Nutrients journal holds the top position, while the Food and Function journal is ranked last.

The co‐citation analysis of journals is employed to examine the relationship between publications within a particular research field (Donthu et al., 2021). Figure 7c represents a visualization map of co‐cited sources. A total of 138 sources out of 223 meet the threshold value of having at least 20 citations. The results yielded four clusters, each comprising distinct colors, i.e., red (66), blue (39), green (26), and yellow (7).

### 3.8 Keyword analysis

**FIGURE 8:**
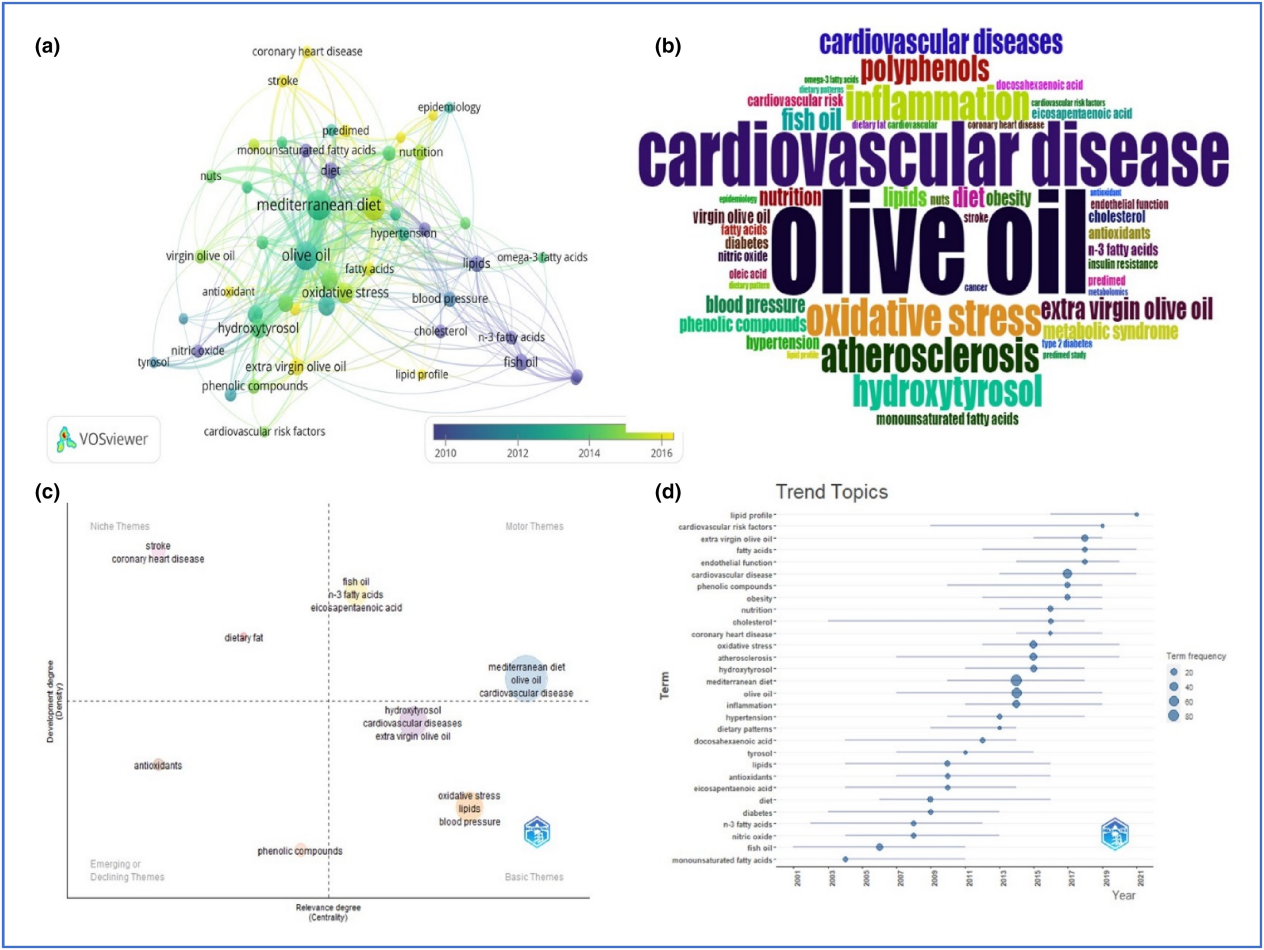
(a) Keyword co‐occurrence overlay visualization map; (b) Word cloud of the author's keywords; (c) Thematic map of keywords; (d) Trend topic on the basis of keywords related to the studies of olive oil research and CVDs.

Keyword analysis offers a detailed explanation of the scope, sub‐categories, subject, and main themes within a given scientific domain. Additionally, authors often include keywords, also known as author keywords, in order to explain the primary concepts of their research (Nobanee et al., 2021). Furthermore, this analysis can also provide the basis for correlation among the author's keywords and their use in identifying the most significant documents in the field of olive oil and CVD research. The co‐occurrence analysis of keywords in the present study showed that there are a total of 1016 author's keywords, out of which only 50 met the threshold for a minimum occurrence of five times. Figure 8a shows the overlay visualization analysis map of the author's keywords. The presented map illustrates five distinct clusters. The initial cluster (*n* = 20) consists of the keywords “cardiovascular disease,” “Mediterranean diet,” “diabetes,” “obesity,” and “olive oil.” The second cluster (*n* = 12) is composed of keywords related to the biological properties of olive oil, such as “antioxidant” and “insulin resistance.” The third (*n* = 8), fourth (*n* = 8), and fifth (*n* = 2) clusters encompass the chemical constituents, applications, and dietary patterns of olive oil, respectively.

In this study, a word cloud of 50 keywords related to olive oil and CVD research was also extracted from the Scopus database, as depicted in Figure 8b. Word clouds offer viewers a visual overview of words that are extensively employed; the larger and bolder the term is highlighted, the more frequently it appears in texts (Uyanga et al., 2023). The keywords with maximum frequency are “Mediterranean diet” (84) and “olive oil” (74), indicating a significant association between the two, as olive oil holds a crucial role in the Mediterranean diet (Huang & Sumpio, 2008; Uylaşer & Yildiz, 2014). Another frequently occurring keyword is “cardiovascular disease” (46), which describes the consumption of olive oil for the prevention of CVDs (Rodríguez et al., 2023; Samakidou et al., 2023; Visioli & Galli, 1998).

The application of thematic maps to depict the conceptual framework within a particular research domain constitutes a fundamental aspect of scientific mapping methodologies (Kemeç & Altınay, 2023). Themes comprise a cluster of keywords that are grouped together within a single circle, arranged by their density and centrality. This arrangement enables the generation of a two‐dimensional visual map (Uyanga et al., 2023). The thematic map depicted in Figure 8c provides a classification of themes based on their respective quadrants. In the upper right quadrant, we observe the presence of motor themes such as “Fish oil”, “Mediterranean diet”, and “Olive oil”. Meanwhile, basic or fundamental themes are positioned in the lower‐right quadrant. The upper‐left quadrant consists of niche themes, while the lower‐left quadrant encompasses themes that are either emerging or declining.

By employing Callon's centrality, Callon's density, rank centrality, and rank density measurements for thematic clusters (as outlined in Table 7), the progression of themes was characterized, demonstrating the multiple evolutionary relationships. These analyses elucidated the field's advancement, developmental stages, evolutionary pathways, and shifts in thematic content over time (Ejaz et al., 2022).

**TABLE 7:** Measurements of thematic clusters for keywords in olive oil research and cardiovascular diseases.

Cluster	Callon centrality	Callon density	Rank centrality	Rank density	Cluster frequency
Dietary fat	0.028	16.67	3	6	6
Mediterranean diet	0.655	13.11	8	5	414
Hydroxytyrosol	0.201	11.48	6	4	78
Oxidative stress	0.370	10.40	7	2	68
Antioxidants	0	11.12	1.5	3	9
Phenolic compounds	0.052	9.10	4	1	11
Stroke	0	17.86	1.5	8	13
Fish oil	0.010	17.85	5	7	40

Figure 8d uses keywords to portray the evolution of research articles throughout the years. The keywords of the articles that were published during the period of 2003–2013 were concentrated on the chemical composition of olive oil and exhibit low term frequency. However, in recent years, there has been a noticeable shift in focus toward the exploration of keywords such as atherosclerosis, cholesterol, and cardiovascular risk factors. This recent shift in trending keywords indicates the correlation of olive oil with CVDs. However, the terms with the highest frequencies were the Mediterranean diet and olive oil.

### 3.9 Co‐cited reference analysis

**FIGURE 9:**
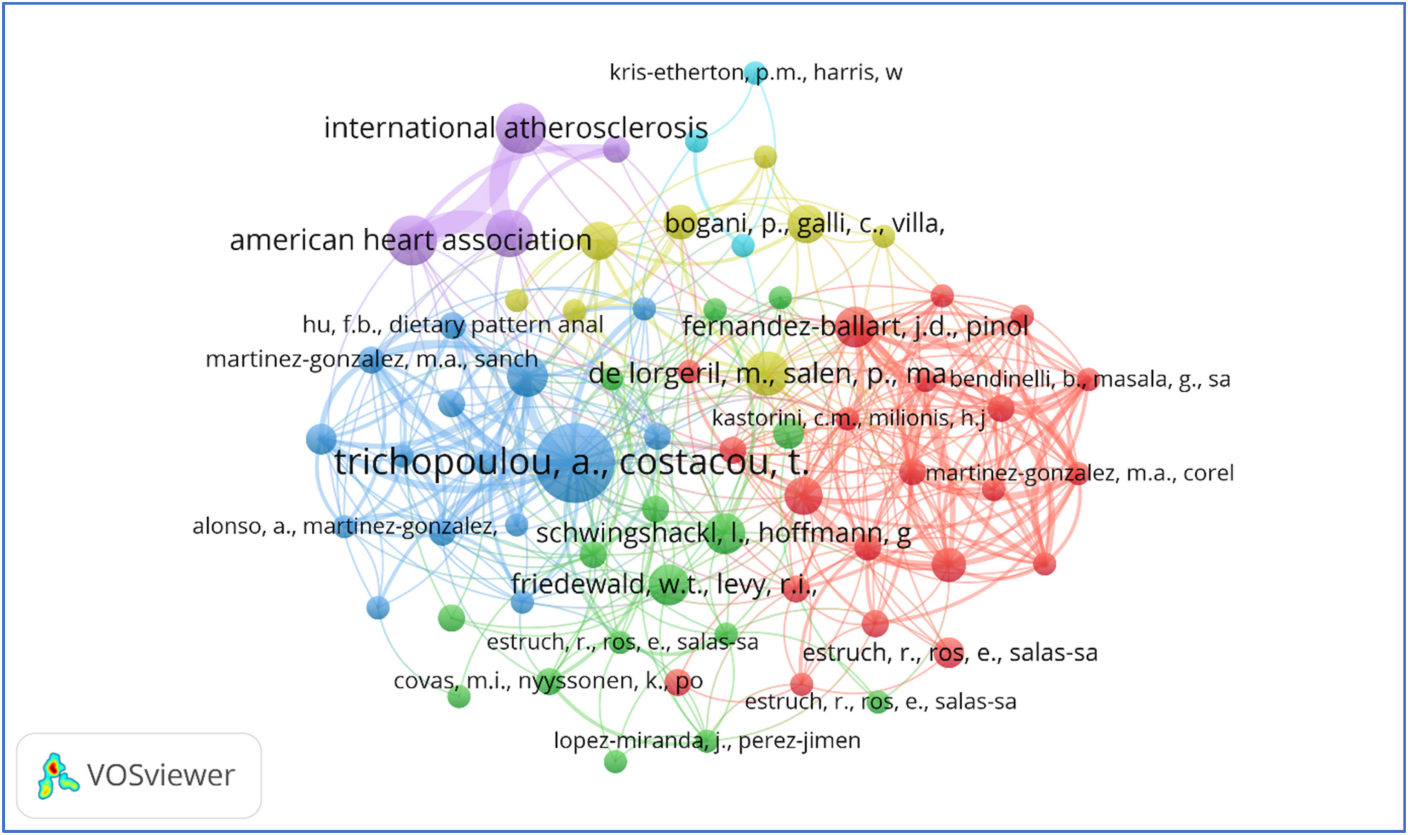
Co‐citation among cited references.

When two or more articles are cited simultaneously, they are considered co‐cited (Suban, 2022). Thus, as per the selected documents for this study, the results of the co‐citation reference analysis are represented in Figure 9. According to VOSviewer analysis, 67 out of 467 cited references meet the threshold value of having a minimum of four citations. A total of six clusters were extracted; nodes of the same color depicted the same cluster of authors. The link between each node (author) represents the co‐citation among them. Additionally, the thickness of the linkage between them represents the frequency at which they are cited (Boyack & Klavans, 2010). The results revealed that the publication titled “Adherence to a Mediterranean diet and survival in a Greek population” (Trichopoulou et al., 2003), published in the “*New England Journal of Medicine*”, appeared as the most cited article with 23 co‐citations and a strength link of 60.

## 4 STRENGTH AND LIMITATIONS

The present study consists of a comprehensive bibliometric analysis dealing with every aspect, including publication trends, contributions of countries, authors, organizations, journals, and keywords in the recent research trends in olive oil and CVDs. The studies, including bibliometric analyses, can effectively update the latest trends and research directions. These tools provide valuable insights into the hot topics within a specific field and enable predictions of future research trends across various aspects. Although analyzing potent keywords is an effective way for researchers to enhance their understanding of the impact of olive oil on CVDs, this approach could potentially lead to the generation of novel ideas that contribute to the field. Furthermore, scholars find it beneficial as they can easily connect with highly productive and renowned authors, institutions, and countries in their specific domains. As an outcome, this kind of investigation offers crucial knowledge for researchers and professionals to comprehend the areas for improvement in this field.

There were a few limitations in this study that might influence the scope and interpretation of the data. These limitations are outlined as follows:

- The study used two different analysis tools, Biblioshiny‐R Studio and VOSviewer, to process the data. However, the consistency of the analysis results obtained from these two tools cannot be guaranteed, and this may affect the overall findings and interpretations.
- The study excludes articles written in languages other than English. This exclusion could introduce source bias, as valuable information from non‐English sources might have been overlooked.
- The analysis focused only on specific aspects of the data, potentially leading to the omission of important details. For this study, we exclusively selected research articles.
- While bibliometric analysis offers valuable insights into publication patterns, citations, and collaborations, it falls short in assessing the actual content and quality of research. It should be complemented with other review methods for a more comprehensive evaluation.

## 5 FUTURE RESEARCH OPPORTUNITIES

The significant contributions from countries, organizations, and authors, including “Spain”, “University of Navarra”, and “Estruch R”, offer valuable recommendations for further research to decrease the cases of CVDs. However, the current bibliometric analysis has also found scope for many opportunities for further advancements in this field, as indicated by the following findings.

More research on the relationship between olive oil consumption and cardiovascular health in different cultural and geographical contexts is expected. We can also explore the variations in intake of olive oil and their impact on cardiovascular health among individuals with diverse gender, age, ethnicity, and health conditions. In summary, we are expecting improvements and advancements in research activities on the impact of olive oil on CVDs.

## 6 CONCLUSION

In recent years, the utilization of olive oil and its derivatives in daily life has been characterized due to its nutritional and therapeutic effects. Numerous studies have demonstrated the advantageous effects of olive oil and its metabolites in the management of CVDs. In the present study, a bibliometric analysis and a systematic review of recent research trends regarding the correlation between olive oil and CVD have been conducted based on the selection of 429 published papers. The key outcomes and conclusion of this study are as follows:

- The analysis of affiliations reveals that “Spain” is the primary contributor to the research in this area. Furthermore, the “University of Navarra”, “University of Barcelona”, and “Instituto de Salud Carlos” are the top three research centers that have made significant contributions to this field.
- “Estruch R”, “Salas‐Salvadó J”, and “Ros E” are the three most productive authors in terms of publication count.
- The leading publications in the field are “*Nutrients*” and “*American Journal of Clinical Nutrition*”. In accordance with Bradford's law, it has been observed that 16 sources account for one‐third of the 429 publications in this domain.
- Based on keyword co‐occurrence analysis, “Mediterranean diet,” “Olive oil,” “Cardiovascular disease,” “Oxidative stress,” and “Atherosclerosis” were the trending topics in this field.

However, in future research, it would be interesting to employ more bibliometric tools, like CiteSpace or any other analysis methods, to provide a more comprehensive representation of the systematic literature review within this field.

## AUTHOR CONTRIBUTIONS

**Baby Gargi:** Conceptualization (equal); investigation (equal). **Sakshi Painuli:** Conceptualization (supporting); investigation (supporting). **Prabhakar Semwal:** Conceptualization (supporting); formal analysis (equal); methodology (equal); writing – original draft (equal); writing – review and editing (equal). **Deependra Pratap Singh:** Data curation (supporting); formal analysis (equal); methodology (equal). **Rohit Sharma:** Conceptualization (equal); data curation (equal); formal analysis (equal); methodology (equal). **Abdur Rauf:** Conceptualization (equal); formal analysis (equal); methodology (equal); writing – original draft (equal); writing – review and editing (equal). **Anees Ahmed Khalil:** Conceptualization (equal); formal analysis (equal); methodology (equal); writing – original draft (equal); writing – review and editing (equal). **Ahood Khalid:** Conceptualization (equal); formal analysis (supporting); investigation (supporting); methodology (supporting); writing – original draft (supporting); writing – review and editing (supporting). **Hassan A. Hemeg:** Data curation (equal); investigation (equal); project administration (supporting). **Polrat Wilairatana:** Conceptualization (equal); data curation (equal); investigation (equal); methodology (equal); validation (equal); writing – review and editing (equal).

## CONFLICT OF INTEREST STATEMENT

The authors declare no conflict of interest.

## Data Availability

Not applicable.
